# Supplementary material for: Pyruvate Dehydrogenase Kinase 1 inhibition mediated oxidative phosphorylation enhancement in cartilage promotes osteoarthritis progression
Source: BMC Musculoskelet Disord. 2023 Jul 20;24:597. doi: 10.1186/s12891-023-06585-6 (PMC10357736; doi:10.1186/s12891-023-06585-6)
Supplement: Supplementary file 1 — Additional file 1. [file 12891_2023_6585_MOESM1_ESM.zip › MF.Gsea.1653627316242/GOMF_ISOMERASE_ACTIVITY.html]

Details for gene set GOMF\_ISOMERASE\_ACTIVITY[GSEA]

|  || Dataset | 55457\_collapsed\_to\_symbols.cls.cls #OA\_versus\_normal.cls.cls #OA\_versus\_normal\_repos |
| Phenotype | cls.cls#OA\_versus\_normal\_repos |
| Upregulated in class | OA |
| GeneSet | GOMF\_ISOMERASE\_ACTIVITY |
| Enrichment Score (ES) | 0.24766153 |
| Normalized Enrichment Score (NES) | 1.1836077 |
| Nominal p-value | 0.10060976 |
| FDR q-value | 0.5798333 |
| FWER p-Value | 1.0 |
Table: GSEA Results Summary

  

Fig 1: Enrichment plot: GOMF\_ISOMERASE\_ACTIVITY      
 Profile of the Running ES Score & Positions of GeneSet Members on the Rank Ordered List

  

| SYMBOL | TITLE | RANK IN GENE LIST | RANK METRIC SCORE | RUNNING ES | CORE ENRICHMENT || 1 | CRELD2 | cysteine rich with EGF like domains 2 [Source:HGNC Symbol;Acc:HGNC:28150] | 146 | 0.867 | 0.0101 | Yes |
| 2 | GPI | glucose-6-phosphate isomerase [Source:HGNC Symbol;Acc:HGNC:4458] | 166 | 0.842 | 0.0296 | Yes |
| 3 | MPI | mannose phosphate isomerase [Source:HGNC Symbol;Acc:HGNC:7216] | 255 | 0.781 | 0.0422 | Yes |
| 4 | RPE65 | retinoid isomerohydrolase RPE65 [Source:HGNC Symbol;Acc:HGNC:10294] | 308 | 0.743 | 0.0566 | Yes |
| 5 | PDIA5 | protein disulfide isomerase family A member 5 [Source:HGNC Symbol;Acc:HGNC:24811] | 324 | 0.735 | 0.0737 | Yes |
| 6 | PPIE | peptidylprolyl isomerase E [Source:HGNC Symbol;Acc:HGNC:9258] | 334 | 0.733 | 0.0913 | Yes |
| 7 | PGAM1 | phosphoglycerate mutase 1 [Source:HGNC Symbol;Acc:HGNC:8888] | 341 | 0.727 | 0.1089 | Yes |
| 8 | MIF | macrophage migration inhibitory factor [Source:HGNC Symbol;Acc:HGNC:7097] | 555 | 0.640 | 0.1081 | Yes |
| 9 | ENOX1 | ecto-NOX disulfide-thiol exchanger 1 [Source:HGNC Symbol;Acc:HGNC:25474] | 558 | 0.638 | 0.1238 | Yes |
| 10 | HPGDS | hematopoietic prostaglandin D synthase [Source:HGNC Symbol;Acc:HGNC:17890] | 605 | 0.622 | 0.1357 | Yes |
| 11 | ECI2 | enoyl-CoA delta isomerase 2 [Source:HGNC Symbol;Acc:HGNC:14601] | 669 | 0.601 | 0.1457 | Yes |
| 12 | ERP29 | endoplasmic reticulum protein 29 [Source:HGNC Symbol;Acc:HGNC:13799] | 706 | 0.592 | 0.1576 | Yes |
| 13 | AMACR | alpha-methylacyl-CoA racemase [Source:HGNC Symbol;Acc:HGNC:451] | 749 | 0.581 | 0.1688 | Yes |
| 14 | P4HB | prolyl 4-hydroxylase subunit beta [Source:HGNC Symbol;Acc:HGNC:8548] | 777 | 0.575 | 0.1810 | Yes |
| 15 | GSTZ1 | glutathione S-transferase zeta 1 [Source:HGNC Symbol;Acc:HGNC:4643] | 974 | 0.523 | 0.1787 | Yes |
| 16 | DDT | D-dopachrome tautomerase [Source:HGNC Symbol;Acc:HGNC:2732] | 1094 | 0.498 | 0.1818 | Yes |
| 17 | ERP44 | endoplasmic reticulum protein 44 [Source:HGNC Symbol;Acc:HGNC:18311] | 1175 | 0.481 | 0.1875 | Yes |
| 18 | FKBP11 | FKBP prolyl isomerase 11 [Source:HGNC Symbol;Acc:HGNC:18624] | 1200 | 0.477 | 0.1975 | Yes |
| 19 | HSD17B4 | hydroxysteroid 17-beta dehydrogenase 4 [Source:HGNC Symbol;Acc:HGNC:5213] | 1354 | 0.450 | 0.1967 | Yes |
| 20 | ECI1 | enoyl-CoA delta isomerase 1 [Source:HGNC Symbol;Acc:HGNC:2703] | 1453 | 0.435 | 0.1999 | Yes |
| 21 | SPAST | spastin [Source:HGNC Symbol;Acc:HGNC:11233] | 1502 | 0.429 | 0.2068 | Yes |
| 22 | PPIH | peptidylprolyl isomerase H [Source:HGNC Symbol;Acc:HGNC:14651] | 1555 | 0.419 | 0.2131 | Yes |
| 23 | FKBP3 | FKBP prolyl isomerase 3 [Source:HGNC Symbol;Acc:HGNC:3719] | 1566 | 0.418 | 0.2228 | Yes |
| 24 | AIP | aryl hydrocarbon receptor interacting protein [Source:HGNC Symbol;Acc:HGNC:358] | 1649 | 0.406 | 0.2265 | Yes |
| 25 | FKBP2 | FKBP prolyl isomerase 2 [Source:HGNC Symbol;Acc:HGNC:3718] | 1785 | 0.387 | 0.2255 | Yes |
| 26 | GNPDA1 | glucosamine-6-phosphate deaminase 1 [Source:HGNC Symbol;Acc:HGNC:4417] | 1795 | 0.385 | 0.2344 | Yes |
| 27 | PPIC | peptidylprolyl isomerase C [Source:HGNC Symbol;Acc:HGNC:9256] | 1823 | 0.381 | 0.2418 | Yes |
| 28 | ECH1 | enoyl-CoA hydratase 1 [Source:HGNC Symbol;Acc:HGNC:3149] | 1901 | 0.373 | 0.2450 | Yes |
| 29 | PDIA6 | protein disulfide isomerase family A member 6 [Source:HGNC Symbol;Acc:HGNC:30168] | 2073 | 0.353 | 0.2404 | Yes |
| 30 | PIN1 | "peptidylprolyl cis/trans isomerase, NIMA-interacting 1 [Source:HGNC Symbol;Acc:HGNC:8988]" | 2257 | 0.331 | 0.2344 | Yes |
| 31 | FKBP1A | FKBP prolyl isomerase 1A [Source:HGNC Symbol;Acc:HGNC:3711] | 2293 | 0.328 | 0.2398 | Yes |
| 32 | FKBP15 | FKBP prolyl isomerase family member 15 [Source:HGNC Symbol;Acc:HGNC:23397] | 2304 | 0.326 | 0.2471 | Yes |
| 33 | BPGM | bisphosphoglycerate mutase [Source:HGNC Symbol;Acc:HGNC:1093] | 2398 | 0.315 | 0.2477 | Yes |
| 34 | PTGIS | prostaglandin I2 synthase [Source:HGNC Symbol;Acc:HGNC:9603] | 2654 | 0.284 | 0.2348 | No |
| 35 | PIGK | phosphatidylinositol glycan anchor biosynthesis class K [Source:HGNC Symbol;Acc:HGNC:8965] | 2801 | 0.270 | 0.2301 | No |
| 36 | KATNA1 | katanin catalytic subunit A1 [Source:HGNC Symbol;Acc:HGNC:6216] | 2891 | 0.261 | 0.2296 | No |
| 37 | RPUSD2 | RNA pseudouridine synthase domain containing 2 [Source:HGNC Symbol;Acc:HGNC:24180] | 2934 | 0.257 | 0.2327 | No |
| 38 | FKBP1B | FKBP prolyl isomerase 1B [Source:HGNC Symbol;Acc:HGNC:3712] | 2966 | 0.254 | 0.2366 | No |
| 39 | PGAM2 | phosphoglycerate mutase 2 [Source:HGNC Symbol;Acc:HGNC:8889] | 3008 | 0.249 | 0.2396 | No |
| 40 | RPE | ribulose-5-phosphate-3-epimerase [Source:HGNC Symbol;Acc:HGNC:10293] | 3153 | 0.235 | 0.2342 | No |
| 41 | FKBP10 | FKBP prolyl isomerase 10 [Source:HGNC Symbol;Acc:HGNC:18169] | 3345 | 0.217 | 0.2246 | No |
| 42 | MMUT | methylmalonyl-CoA mutase [Source:HGNC Symbol;Acc:HGNC:7526] | 3362 | 0.215 | 0.2287 | No |
| 43 | PPIB | peptidylprolyl isomerase B [Source:HGNC Symbol;Acc:HGNC:9255] | 3372 | 0.214 | 0.2334 | No |
| 44 | TXNDC5 | thioredoxin domain containing 5 [Source:HGNC Symbol;Acc:HGNC:21073] | 3378 | 0.214 | 0.2383 | No |
| 45 | TOP2B | DNA topoisomerase II beta [Source:HGNC Symbol;Acc:HGNC:11990] | 3458 | 0.206 | 0.2372 | No |
| 46 | HSD3B1 | "hydroxy-delta-5-steroid dehydrogenase, 3 beta- and steroid delta-isomerase 1 [Source:HGNC Symbol;Acc:HGNC:5217]" | 3695 | 0.186 | 0.2234 | No |
| 47 | PDIA4 | protein disulfide isomerase family A member 4 [Source:HGNC Symbol;Acc:HGNC:30167] | 3756 | 0.181 | 0.2232 | No |
| 48 | FKBP14 | FKBP prolyl isomerase 14 [Source:HGNC Symbol;Acc:HGNC:18625] | 3825 | 0.174 | 0.2222 | No |
| 49 | EBP | EBP cholestenol delta-isomerase [Source:HGNC Symbol;Acc:HGNC:3133] | 3848 | 0.171 | 0.2247 | No |
| 50 | PGM1 | phosphoglucomutase 1 [Source:HGNC Symbol;Acc:HGNC:8905] | 3990 | 0.157 | 0.2176 | No |
| 51 | TPI1 | triosephosphate isomerase 1 [Source:HGNC Symbol;Acc:HGNC:12009] | 4059 | 0.150 | 0.2160 | No |
| 52 | PBLD | phenazine biosynthesis like protein domain containing [Source:HGNC Symbol;Acc:HGNC:23301] | 4197 | 0.138 | 0.2087 | No |
| 53 | PMM1 | phosphomannomutase 1 [Source:HGNC Symbol;Acc:HGNC:9114] | 4238 | 0.134 | 0.2089 | No |
| 54 | TBXAS1 | thromboxane A synthase 1 [Source:HGNC Symbol;Acc:HGNC:11609] | 4338 | 0.125 | 0.2043 | No |
| 55 | SRR | serine racemase [Source:HGNC Symbol;Acc:HGNC:14398] | 4397 | 0.121 | 0.2027 | No |
| 56 | PTGES | prostaglandin E synthase [Source:HGNC Symbol;Acc:HGNC:9599] | 4399 | 0.121 | 0.2057 | No |
| 57 | HSD3B2 | "hydroxy-delta-5-steroid dehydrogenase, 3 beta- and steroid delta-isomerase 2 [Source:HGNC Symbol;Acc:HGNC:5218]" | 4424 | 0.119 | 0.2068 | No |
| 58 | FKBP9 | FKBP prolyl isomerase 9 [Source:HGNC Symbol;Acc:HGNC:3725] | 4541 | 0.110 | 0.2004 | No |
| 59 | PPID | peptidylprolyl isomerase D [Source:HGNC Symbol;Acc:HGNC:9257] | 5029 | 0.071 | 0.1641 | No |
| 60 | LSS | lanosterol synthase [Source:HGNC Symbol;Acc:HGNC:6708] | 5182 | 0.058 | 0.1536 | No |
| 61 | PGM3 | phosphoglucomutase 3 [Source:HGNC Symbol;Acc:HGNC:8907] | 5290 | 0.049 | 0.1464 | No |
| 62 | CRELD1 | cysteine rich with EGF like domains 1 [Source:HGNC Symbol;Acc:HGNC:14630] | 5555 | 0.028 | 0.1265 | No |
| 63 | PIN4 | "peptidylprolyl cis/trans isomerase, NIMA-interacting 4 [Source:HGNC Symbol;Acc:HGNC:8992]" | 5567 | 0.027 | 0.1263 | No |
| 64 | PTGDS | prostaglandin D2 synthase [Source:HGNC Symbol;Acc:HGNC:9592] | 5583 | 0.025 | 0.1257 | No |
| 65 | ENOPH1 | enolase-phosphatase 1 [Source:HGNC Symbol;Acc:HGNC:24599] | 5615 | 0.023 | 0.1239 | No |
| 66 | RENBP | renin binding protein [Source:HGNC Symbol;Acc:HGNC:9959] | 5653 | 0.019 | 0.1215 | No |
| 67 | HYI | hydroxypyruvate isomerase (putative) [Source:HGNC Symbol;Acc:HGNC:26948] | 5777 | 0.010 | 0.1121 | No |
| 68 | IDI1 | isopentenyl-diphosphate delta isomerase 1 [Source:HGNC Symbol;Acc:HGNC:5387] | 5842 | 0.006 | 0.1072 | No |
| 69 | ISYNA1 | inositol-3-phosphate synthase 1 [Source:HGNC Symbol;Acc:HGNC:29821] | 5973 | -0.003 | 0.0971 | No |
| 70 | ITPK1 | inositol-tetrakisphosphate 1-kinase [Source:HGNC Symbol;Acc:HGNC:6177] | 6095 | -0.012 | 0.0879 | No |
| 71 | GLRX2 | glutaredoxin 2 [Source:HGNC Symbol;Acc:HGNC:16065] | 6116 | -0.014 | 0.0867 | No |
| 72 | PTPA | protein phosphatase 2 phosphatase activator [Source:HGNC Symbol;Acc:HGNC:9308] | 6120 | -0.014 | 0.0868 | No |
| 73 | QSOX1 | quiescin sulfhydryl oxidase 1 [Source:HGNC Symbol;Acc:HGNC:9756] | 6130 | -0.015 | 0.0865 | No |
| 74 | GLCE | glucuronic acid epimerase [Source:HGNC Symbol;Acc:HGNC:17855] | 6148 | -0.016 | 0.0855 | No |
| 75 | PTGES2 | prostaglandin E synthase 2 [Source:HGNC Symbol;Acc:HGNC:17822] | 6269 | -0.025 | 0.0768 | No |
| 76 | PUS7L | pseudouridine synthase 7 like [Source:HGNC Symbol;Acc:HGNC:25276] | 6299 | -0.027 | 0.0752 | No |
| 77 | TOP2A | DNA topoisomerase II alpha [Source:HGNC Symbol;Acc:HGNC:11989] | 6377 | -0.034 | 0.0700 | No |
| 78 | PDIA3 | protein disulfide isomerase family A member 3 [Source:HGNC Symbol;Acc:HGNC:4606] | 6607 | -0.050 | 0.0533 | No |
| 79 | PPIAL4G | peptidylprolyl isomerase A like 4G [Source:HGNC Symbol;Acc:HGNC:33996] | 6779 | -0.061 | 0.0415 | No |
| 80 | GFUS | GDP-L-fucose synthase [Source:HGNC Symbol;Acc:HGNC:12390] | 7317 | -0.103 | 0.0020 | No |
| 81 | SPO11 | SPO11 initiator of meiotic double stranded breaks [Source:HGNC Symbol;Acc:HGNC:11250] | 7436 | -0.114 | -0.0044 | No |
| 82 | DHRS9 | dehydrogenase/reductase 9 [Source:HGNC Symbol;Acc:HGNC:16888] | 7492 | -0.118 | -0.0058 | No |
| 83 | DSE | dermatan sulfate epimerase [Source:HGNC Symbol;Acc:HGNC:21144] | 7493 | -0.118 | -0.0029 | No |
| 84 | ALOXE3 | arachidonate lipoxygenase 3 [Source:HGNC Symbol;Acc:HGNC:13743] | 7502 | -0.119 | -0.0005 | No |
| 85 | AIPL1 | aryl hydrocarbon receptor interacting protein like 1 [Source:HGNC Symbol;Acc:HGNC:359] | 7914 | -0.148 | -0.0290 | No |
| 86 | EHHADH | enoyl-CoA hydratase and 3-hydroxyacyl CoA dehydrogenase [Source:HGNC Symbol;Acc:HGNC:3247] | 7968 | -0.153 | -0.0293 | No |
| 87 | PTGES3 | prostaglandin E synthase 3 [Source:HGNC Symbol;Acc:HGNC:16049] | 8802 | -0.217 | -0.0891 | No |
| 88 | DEGS1 | "delta 4-desaturase, sphingolipid 1 [Source:HGNC Symbol;Acc:HGNC:13709]" | 8885 | -0.223 | -0.0900 | No |
| 89 | FKBP8 | FKBP prolyl isomerase 8 [Source:HGNC Symbol;Acc:HGNC:3724] | 8928 | -0.226 | -0.0877 | No |
| 90 | PDIA2 | protein disulfide isomerase family A member 2 [Source:HGNC Symbol;Acc:HGNC:14180] | 8961 | -0.229 | -0.0845 | No |
| 91 | PPWD1 | peptidylprolyl isomerase domain and WD repeat containing 1 [Source:HGNC Symbol;Acc:HGNC:28954] | 9069 | -0.237 | -0.0869 | No |
| 92 | PPIL2 | peptidylprolyl isomerase like 2 [Source:HGNC Symbol;Acc:HGNC:9261] | 9148 | -0.244 | -0.0870 | No |
| 93 | FKBP4 | FKBP prolyl isomerase 4 [Source:HGNC Symbol;Acc:HGNC:3720] | 9205 | -0.248 | -0.0852 | No |
| 94 | THAP4 | THAP domain containing 4 [Source:HGNC Symbol;Acc:HGNC:23187] | 9330 | -0.258 | -0.0885 | No |
| 95 | PMM2 | phosphomannomutase 2 [Source:HGNC Symbol;Acc:HGNC:9115] | 9408 | -0.265 | -0.0879 | No |
| 96 | ALOX12B | "arachidonate 12-lipoxygenase, 12R type [Source:HGNC Symbol;Acc:HGNC:430]" | 9617 | -0.284 | -0.0971 | No |
| 97 | CYP2J2 | cytochrome P450 family 2 subfamily J member 2 [Source:HGNC Symbol;Acc:HGNC:2634] | 9642 | -0.287 | -0.0918 | No |
| 98 | RPIA | ribose 5-phosphate isomerase A [Source:HGNC Symbol;Acc:HGNC:10297] | 9793 | -0.301 | -0.0961 | No |
| 99 | PGM5 | phosphoglucomutase 5 [Source:HGNC Symbol;Acc:HGNC:8908] | 9836 | -0.305 | -0.0918 | No |
| 100 | NAXE | NAD(P)HX epimerase [Source:HGNC Symbol;Acc:HGNC:18453] | 9994 | -0.320 | -0.0961 | No |
| 101 | PPIF | peptidylprolyl isomerase F [Source:HGNC Symbol;Acc:HGNC:9259] | 9999 | -0.321 | -0.0884 | No |
| 102 | SREBF2 | sterol regulatory element binding transcription factor 2 [Source:HGNC Symbol;Acc:HGNC:11290] | 10070 | -0.328 | -0.0858 | No |
| 103 | ITGB3 | integrin subunit beta 3 [Source:HGNC Symbol;Acc:HGNC:6156] | 10310 | -0.352 | -0.0957 | No |
| 104 | DKC1 | dyskerin pseudouridine synthase 1 [Source:HGNC Symbol;Acc:HGNC:2890] | 10591 | -0.382 | -0.1081 | No |
| 105 | CFTR | CF transmembrane conductance regulator [Source:HGNC Symbol;Acc:HGNC:1884] | 10920 | -0.420 | -0.1233 | No |
| 106 | DCT | dopachrome tautomerase [Source:HGNC Symbol;Acc:HGNC:2709] | 10995 | -0.429 | -0.1184 | No |
| 107 | FKBP6 | FKBP prolyl isomerase family member 6 (inactive) [Source:HGNC Symbol;Acc:HGNC:3722] | 11173 | -0.454 | -0.1210 | No |
| 108 | GALE | UDP-galactose-4-epimerase [Source:HGNC Symbol;Acc:HGNC:4116] | 11227 | -0.461 | -0.1137 | No |
| 109 | PUS7 | pseudouridine synthase 7 [Source:HGNC Symbol;Acc:HGNC:26033] | 11421 | -0.490 | -0.1166 | No |
| 110 | HSPD1 | heat shock protein family D (Hsp60) member 1 [Source:HGNC Symbol;Acc:HGNC:5261] | 11427 | -0.492 | -0.1047 | No |
| 111 | PUS3 | pseudouridine synthase 3 [Source:HGNC Symbol;Acc:HGNC:25461] | 11503 | -0.501 | -0.0981 | No |
| 112 | FKBP5 | FKBP prolyl isomerase 5 [Source:HGNC Symbol;Acc:HGNC:3721] | 11536 | -0.505 | -0.0881 | No |
| 113 | TOP3B | DNA topoisomerase III beta [Source:HGNC Symbol;Acc:HGNC:11993] | 11555 | -0.508 | -0.0768 | No |
| 114 | GSTA1 | glutathione S-transferase alpha 1 [Source:HGNC Symbol;Acc:HGNC:4626] | 11608 | -0.517 | -0.0680 | No |
| 115 | ENOSF1 | enolase superfamily member 1 [Source:HGNC Symbol;Acc:HGNC:30365] | 11640 | -0.524 | -0.0574 | No |
| 116 | PPIL6 | peptidylprolyl isomerase like 6 [Source:HGNC Symbol;Acc:HGNC:21557] | 11756 | -0.541 | -0.0529 | No |
| 117 | TOP1 | DNA topoisomerase I [Source:HGNC Symbol;Acc:HGNC:11986] | 11828 | -0.555 | -0.0447 | No |
| 118 | PPIA | peptidylprolyl isomerase A [Source:HGNC Symbol;Acc:HGNC:9253] | 11986 | -0.591 | -0.0423 | No |
| 119 | PUS1 | pseudouridine synthase 1 [Source:HGNC Symbol;Acc:HGNC:15508] | 12260 | -0.664 | -0.0471 | No |
| 120 | PPIG | peptidylprolyl isomerase G [Source:HGNC Symbol;Acc:HGNC:14650] | 12358 | -0.695 | -0.0374 | No |
| 121 | NKTR | natural killer cell triggering receptor [Source:HGNC Symbol;Acc:HGNC:7833] | 12518 | -0.757 | -0.0310 | No |
| 122 | GNE | glucosamine (UDP-N-acetyl)-2-epimerase/N-acetylmannosamine kinase [Source:HGNC Symbol;Acc:HGNC:23657] | 12538 | -0.767 | -0.0134 | No |
| 123 | RANBP2 | RAN binding protein 2 [Source:HGNC Symbol;Acc:HGNC:9848] | 12609 | -0.804 | 0.0011 | No |
| 124 | TOP3A | DNA topoisomerase III alpha [Source:HGNC Symbol;Acc:HGNC:11992] | 12707 | -0.867 | 0.0151 | No |
Table: GSEA details [plain text format]

  

Fig 2: GOMF\_ISOMERASE\_ACTIVITY      
 Blue-Pink O' Gram in the Space of the Analyzed GeneSet

  

Fig 3: GOMF\_ISOMERASE\_ACTIVITY: Random ES distribution      
 Gene set null distribution of ES for **GOMF\_ISOMERASE\_ACTIVITY**
